# The association between adherence to the EAT-Lancet diet and cognitive ageing

**DOI:** 10.1093/ageing/afae032

**Published:** 2024-05-15

**Authors:** Annick P M van Soest, Ondine van de Rest, Renger F Witkamp, Lisette C P G M de Groot

**Affiliations:** Division of Human Nutrition and Health, Wageningen University & Research, Wageningen, The Netherlands; Division of Human Nutrition and Health, Wageningen University & Research, Wageningen, The Netherlands; Division of Human Nutrition and Health, Wageningen University & Research, Wageningen, The Netherlands; Division of Human Nutrition and Health, Wageningen University & Research, Wageningen, The Netherlands

**Keywords:** planetary health diet, plant-based diet, nutrition, brain ageing, healthy ageing, older people

## Abstract

**Background:**

The EAT-Lancet commission has proposed a dietary pattern that is both sustainable and healthy. However, the impact of this diet on cognition in older adults remains unexplored. Therefore, we examined the association between adherence to the EAT-Lancet diet and cognitive ageing.

**Methods:**

We used data from a previous intervention study involving cognitively healthy community-dwelling adults aged ≥65 years. Adherence to the EAT-Lancet diet was calculated using a recently published index and a 190-item food frequency questionnaire. Global and domain-specific cognitive functioning were assessed at baseline and after 2 years using a neuropsychological test battery. Multivariate-adjusted linear regression was conducted to examine associations between EAT-Lancet diet adherence and cognitive functioning (*n* = 630) and 2-year change (*n* = 302).

**Results:**

Greater adherence to the EAT-Lancet diet was associated with better global cognitive functioning (β per SD = 3.7 points [95% CI]: 0.04 [0.00, 0.08]) and slower rate of decline (β per SD [95% CI]: 0.05 [0.02, 0.08]). With respect to domain-specific functioning, beneficial associations were observed cross-sectionally for executive functioning (*P* < 0.01), and longitudinally for change in executive functioning (*P* < 0.01) and attention and working memory (*P* < 0.01). The degree of adherence to the EAT-Lancet was not associated with (changes in) information processing speed or episodic memory.

**Conclusion:**

We demonstrated that greater adherence to the EAT-Lancet diet is associated with better global cognitive functioning and slower cognitive decline among cognitively healthy older adults. Further research is needed to confirm these findings and assess the potential benefits of the EAT-Lancet diet for the ageing population in a broader context.

## Key Points

- This study explores the association between the EAT-Lancet diet, a healthy and sustainable dietary pattern, and cognitive ageing.
- EAT-Lancet diet adherence was positively associated with global cognition and slower decline in cognitively healthy older adults.
- Replication of these findings and extension to other outcomes would be required to verify the relevance to the ageing population.

## Introduction

The diet we consume has a major impact on both human and environmental health. Poor quality diets, such as the Western diet, are associated with an increased risk of chronic diseases [1]. Simultaneously, food production is among the most important drivers of global environmental change, depletion of water and biodiversity loss [2]. Consequently, there is an urgent need for diets that promote both human and environmental health.

The EAT-Lancet commission has proposed the EAT-Lancet diet, a healthy reference diet that fits within planetary boundaries [3]. This diet was developed based on extensive literature review and consists of a large variety in plant foods with few foods from animal sources. Specifically, intakes of vegetables, fruits, legumes, whole grains, nuts and fish are emphasised. Eggs, poultry and dairy are considered optional foods that should be consumed in moderation, and intake of red meat and starchy vegetables should be minimised [3].

While a substantial body of literature supports the health benefits of individual components of the EAT-Lancet diet, the diet as a whole has only been studied for a limited number of health domains. For example, studies have demonstrated that better adherence to the EAT-Lancet diet is associated with lower risk of mortality [4] and type 2 diabetes [5–7], as well as favourable cardiovascular [8–10] and anthropometric [11, 12] outcomes. Yet, the probable benefits of the EAT-Lancet diet on healthy ageing, including the effect on cognitive abilities, have not been studied.

Therefore, the current study aims to investigate the association between adherence to the EAT-Lancet diet and cognitive functioning and 2-year cognitive decline in healthy older adults without cognitive complaints.

## Methods

### Participants and study design

We made use from data of the B-proof study, a randomised double-blind controlled trial on the effect of B-vitamin supplementation (400 μg folic acid and 500 μg vitamin B_12_) versus placebo for 2 years on fracture incidence. Cognitive functioning was measured as secondary outcome. Detailed information on study design and participants has been described previously [13]. In short, participants were community-dwelling older adults aged 65 years and older with elevated homocysteine levels (12–50 μmol/L). None of the participants had a renal insufficiency (creatinine >150 μmol/L) or a cancer diagnosis in the past 5 years. In the present study, we performed a cross-sectional analysis using baseline data and a longitudinal analysis spanning 2 years, using data from the control group only. Follow-up data from the intervention group was excluded to eliminate potential interference from the B-vitamin supplementation. While the B-vitamin intervention was not effective in slowing cognitive decline in the complete B-proof study population [14], a *post hoc* analysis indicated that the B-vitamins may have slowed cognitive ageing in a subgroup [15]. Data were collected between October 2008 and March 2013 at three locations in the Netherlands: Erasmus Medical Center, VU University Medical Center and Wageningen University & Research. The current analysis is based on the Wageningen subgroup only, as extensive dietary and cognitive assessment was only done for Wageningen participants. Of the *n* = 664 participants with available dietary and cognition data, data from 6 participants were excluded due to unreliable energy intake (men <800 kcal or >4,200 kcal, women <500 kcal or >3,500 kcal, *n* = 2), missing baseline cognition (*n* = 4) or missing ApoE genotype (*n* = 28) data. The final study sample comprised *n* = 630 participants. The B-proof trial was approved by the Medical Ethics committee from Wageningen University & Research and was registered under NCT00696514 at clinicaltrials.gov. All participants provided informed consent.

### Dietary assessment

Dietary intake was assessed at baseline by a validated 190-item food frequency questionnaire (FFQ) [16, 17], which covered consumption frequencies in the past month. Standard portion sizes and commonly used household measures were used to estimate portion sizes. Average daily nutrient intakes were computed using data from the Dutch food composition database [18] and an added sugar composition database [19].

As a measure of EAT-Lancet diet adherence, we calculated the EAT-Lancet index based on the methods proposed by Stubbendorff and colleagues [4]. This index is based on 14 food components, divided into 7 ‘emphasised’ food components (vegetables, fruits, unsaturated oils, legumes, nuts, whole grains and fish) and 7 ‘limited’ intake food components (beef and lamb, pork, poultry, eggs, dairy, potatoes and added sugar) (Supplementary Table S1). To calculate the index score, first an energy adjustment was applied according to the nutrient density approach [20]. The intake of a food component in grams was converted to intake in grams per 2,500 kcal, as the EAT-Lancet diet is based on an intake of 2,500 kcal/day. Subsequently, each energy-adjusted food component was assigned a score ranging from 0 to 3 depending on the level of intake according to the cut-offs set by Stubbendorff et al. [4]. For ‘emphasised’ food components, higher intakes received higher scores whereas ‘limited’ food components received lower scores for higher intakes. The scores for the 14 food components were totalled to calculate the EAT-Lancet index, with score 0 representing poorest adherence to score 42 for perfect adherence.

### Cognitive testing

Cognitive functioning was assessed by trained research assistants at baseline and after 2 years. This was done with a comprehensive battery of cognitive tests including six different tests: Rey Auditory Verbal Learning Test (RAVLT) (subtests immediate, delayed and recognition) [21], Digit Span task [22], Trail Making Test (TMT) (part A and B) [23], Stroop Colour-Word test (part I, II and III) [24], Symbol Digit Modalities Test [25] and Letter Fluency [26] (Supplementary Table S2). To limit learning effects, we used parallel versions at year 2 assessment for RAVLT, TMT and Letter Fluency.

To reduce the number of cognition outcomes, raw scores from each test were converted into cognitive composite *Z*-scores using population baseline mean and standard deviation. We reversed the *Z*-scores for the TMT and Stroop Colour-Word test as smaller values indicate better cognition. The individual *Z*-scores per test were combined into composite scores for global and domain-specific cognitive functioning according to the formulas below.


\begin{align*} \mathrm{Global}\ \mathrm{cognition}=&({Z}_{\mathrm{Episodic}\ \mathrm{memory}}\\&+{Z}_{\mathrm{Attention}\ \mathrm{and}\ \mathrm{working}\ \mathrm{memory}}\\&+{Z}_{\mathrm{Information}\ \mathrm{processing}\ \mathrm{speed}}\\&+{Z}_{\mathrm{Executive}\ \mathrm{functioning}})/4 \end{align*}

\begin{align*} \mathrm{Episodic}\ \mathrm{memory}=&({Z}_{\mathrm{RAVLT}\ \mathrm{immediate}}\\&+{Z}_{\mathrm{RAVLT}\ \mathrm{delayed}}\\&+{Z}_{\mathrm{RAVLT}\ \mathrm{recognition}})/3 \end{align*}

\begin{align*} \mathrm{Attention}\ \mathrm{and}\ \mathrm{working}\ \mathrm{memory}=&({Z}_{\mathrm{Digit}\ \mathrm{span}\ \mathrm{forward}}\\&+{Z}_{\mathrm{Digit}\ \mathrm{span}\ \mathrm{backward}})/2 \end{align*}

\begin{align*} \mathrm{Information}\ \mathrm{processing}\ \mathrm{speed}=&(-{Z}_{\mathrm{Stroop}\ \mathrm{mean}\ \mathrm{I}\ \mathrm{and}\ \mathrm{I}\mathrm{I}}\\&+-{Z}_{\mathrm{TMT}\ \mathrm{part}\ \mathrm{A}}\\&+{Z}_{\mathrm{SDMT}})/3 \end{align*}

\begin{align*} \mathrm{Executive}\ \mathrm{functioning}=&(-{Z}_{\mathrm{Stroop}\ \mathrm{interference}}\\&+-{Z}_{\mathrm{TMT}\ \mathrm{part}\ \mathrm{B}/\mathrm{A}}\\&+{Z}_{\mathrm{Fluency}})/3 \end{align*}$$

In addition to the cognitive test battery, Mini Mental State Examination (MMSE) [27] was administered following standardised protocol. This test was assessed for descriptive purposes and serves as an indicator of the participants’ cognitive state.

### Covariates

Trained research assistants collected data on age, sex, education level, smoking status and physical activity [28] via questionnaires. Height and weight were measured following standardised protocol and were used to calculate body mass index (BMI) using the formula weight (kg)/(height (m))^2^. ApoE genotype was determined using TaqMan analysis.

### Statistical analysis

Data are reported as *n* (%), mean (SE) or median (IQR). Differences in baseline characteristics across tertiles were compared using ANOVA for continuous variables and chi-square test for categorical variables. We performed multiple linear regression analysis to examine the association between adherence to the EAT-Lancet diet and global and domain-specific cognitive functioning and decline. For the cross-sectional analysis, we modelled the cognitive composite *Z*-score as a function of EAT-Lancet index score (categorical and continuous) using data from the complete study population. In the longitudinal analysis, the change in cognition *Z*-score between baseline and after 2 years of follow-up was modelled as a function of the EAT-Lancet index score (categorical and continuous) in the control group only. We excluded participants in the B-vitamin intervention group to eliminate influence of the supplementation. We adjusted the final model for the covariates age (years), sex, education (low, middle, high), ApoE4 carrier status, BMI (in kg/m^2^), physical activity (METh/w), smoking (never, former, current) and alcohol intake (light, moderate, excessive). In addition, the longitudinal analysis was adjusted for baseline cognitive composite *Z*-score. A *P*-value smaller than 0.05 was considered significant. All analyses were performed using RStudio version 1.4.0 [29].

## Results

### Baseline characteristics

The average age was 72.1 ± 5.3 years and 60% of the participants were male (Table 1). The mean BMI was 27.2 ± 3.9 kg/m^2^, indicating that on average participants were overweight. In addition, participants were cognitively healthy as demonstrated by a median MMSE of 29 [28–30]. The EAT-Lancet index score ranged from 8 to 33 in the total study population, with scores ranging from 8 to 18 in the low, 19–21 in the middle and 22–33 in the high tertiles. Participants classified in the lowest tertile of EAT-Lancet diet adherence had on average a higher BMI, had a lower level of education, were more often current smoker and scored lower on MMSE compared to individuals who fell into the tertile with highest adherence.

**Table 1:** Baseline characteristics of the B-proof study population per tertile of the EAT-Lancet index

Characteristic	Overall (*n* = 630)	Low (*n* = 241)	Middle (*n* = 203)	High (*n* = 186)	*P*-value
EAT-Lancet index (range)	8–33	8–18	19–21	22–33	<0.01
Age (years)	72.1 ± 5.3	72.2 ± 5.3	72.5 ± 5.2	71.4 ± 5.4	0.11
Sex *n* (%)					0.18
Male	378 (60%)	144 (60%)	131 (65%)	103 (55%)	
Female	252 (40%)	97 (40%)	72 (35%)	83 (45%)	
Level of education *n* (%)					<0.01
Low	268 (43%)	127 (53%)	72 (35%)	69 (37%)	
Middle	151 (24%)	54 (22%)	55 (27%)	42 (23%)	
High	211 (34%)	60 (25%)	76 (37%)	75 (40%)	
ApoE4 carrier	192 (31%)	60 (25%)	67 (33%)	65 (35%)	0.05
BMI (kg/m^2^)	27.2 ± 3.9	27.7 ± 3.8	27.0 ± 4.2	26.7 ± 3.7	0.03
Physical activity (METh/w)	53 (33–80)	51 (31–77)	56 (34–84)	54 (35–77)	0.32
Smoking behaviour *n* (%)					0.01
Current smoker	67 (11%)	35 (15%)	20 (10%)	12 (6%)	
Former smoker	372 (59%)	130 (54%)	133 (66%)	109 (59%)	
Never smoker	191 (30%)	76 (32%)	50 (25%)	65 (35%)	
Alcohol intake *n* (%)					0.99
Light	405 (64%)	157 (65%)	131 (65%)	117 (63%)	
Moderate	204 (32%)	76 (32%)	65 (32%)	63 (34%)	
Excessive	21 (3%)	8 (3%)	7 (3%)	6 (3%)	
MMSE score	29 (28–30)	29 (27–29)	29 (28–30)	29 (28–30)	0.01

*Abbreviations*: BMI, body mass index; MMSE, Mini Mental State Examination. Data are mean ± SD, median (IQR) or number (%).

In addition, nutrient intake differed largely between tertiles (Supplementary Table S3). Participants with higher adherence to the EAT-Lancet diet consumed more plant protein, polyunsaturated fatty acids in general, omega-3 fatty acids and fibres, while their intakes of animal protein, cholesterol and saturated fat were lower.

The EAT-Lancet diet differs from other dietary patterns with demonstrated benefits for the ageing brain (MIND diet, Mediterranean diet) (Supplementary Table S4). To obtain a maximum adherence score, the EAT-Lancet diet allows fewer portions of animal-based foods and requires more portions of plant-based foods.

### Cross-sectional analysis

Higher adherence to the EAT-Lancet diet was associated with better global cognitive functioning (Table 2). For each SD (3.7 points) increase in EAT-Lancet diet score, the global cognition composite increased with 0.04 units (95% CI 0.00, 0.08; *P* = 0.03). However, this finding was not confirmed in the categorical analysis (difference T1 versus T3 [95% CI]: 0.07 [−0.03, 0.16], *P* = 0.17).

**Table 2:** Regression output association EAT-Lancet index and cognitive functioning (cross-sectional)

	Crude model	Model 1	Model 2
Global cognition			
Tertile 1	REF	REF	REF
Tertile 2	0.11 [0.01, 0.21] 0.02	0.06 [−0.04, 0.15] 0.23	0.04 [−0.05, 0.13] 0.37
Tertile 3	0.19 [0.09, 0.29] <0.01	0.08 [−0.01, 0.18] 0.09	0.07 [−0.03, 0.16] 0.17
Continuous	0.10 [0.06, 0.14] <0.01	0.05 [0.01, 0.09] 0.01	0.04 [0.00, 0.08] 0.03
Episodic memory			
Tertile 1	REF	REF	REF
Tertile 2	−0.06 [−0.18, 0.07] 0.39	−0.07 [−0.19, 0.05] 0.27	−0.09 [−0.21, 0.04] 0.17
Tertile 3	0.07 [−0.06, 0.20] 0.27	0.00 [−0.13, 0.12] 0.94	−0.03 [−0.16, 0.09] 0.60
Continuous	0.05 [−0.01, 0.10] 0.08	0.01 [−0.05, 0.06] 0.81	0.00 [−0.06, 0.05] 0.88
Attention and working memory			
Tertile 1	REF	REF	REF
Tertile 2	0.20 [0.04, 0.36] 0.01	0.10 [−0.05, 0.25] 0.20	0.10 [−0.05, 0.26] 0.19
Tertile 3	0.21 [0.05, 0.37] 0.01	0.07 [−0.09, 0.23] 0.37	0.08 [−0.08, 0.24] 0.34
Continuous	0.10 [0.04, 0.17] <0.01	0.03 [−0.03, 0.10] 0.30	0.04 [−0.03, 0.11] 0.23
Information processing speed			
Tertile 1	REF	REF	REF
Tertile 2	0.15 [0.00, 0.29] 0.05	0.11 [−0.03, 0.25] 0.12	0.08 [−0.06, 0.22] 0.24
Tertile 3	0.25 [0.10, 0.40] <0.01	0.15 [0.00, 0.29] 0.04	0.12 [−0.02, 0.26] 0.10
Continuous	0.11 [0.05, 0.18] <0.01	0.07 [0.01, 0.13] 0.02	0.06 [0.00, 0.12] 0.05
Executive functioning			
Tertile 1	REF	REF	REF
Tertile 2	0.17 [0.04, 0.30] 0.01	0.07 [−0.05, 0.20] 0.24	0.06 [−0.07, 0.19] 0.35
Tertile 3	0.20 [0.07, 0.34] <0.01	0.09 [−0.04, 0.22] 0.18	0.07 [−0.06, 0.20] 0.27
Continuous	0.12 [0.07, 0.18] <0.01	0.07 [0.02, 0.12] 0.01	0.07 [0.01, 0.12] 0.02

Model 1: adjusted for age, gender, education and ApoE4 carrier status; Model 2: additionally adjusted for BMI, physical activity, smoking and alcohol consumption. Data are β [95% CI] *P*-value. In the continuous analysis, β is shown per SD = 3.7 points increment in EAT-Lancet index.

With respect to domain-specific cognitive functioning, higher adherence to the EAT-Lancet diet was associated with better executive functioning performance (β per SD [95% CI]: 0.07 [0.01, 0.12], *P* = 0.02), but again this finding was not replicated in the categorical analysis (*P* = 0.27). There was a trend towards an association between information processing speed performance and the level of adherence to the EAT-Lancet diet (β per SD [95% CI]: 0.06 [0.00, 0.12], *P* = 0.05). For episodic memory and attention and working memory, no associations were found between EAT-Lancet diet adherence in both continuous and categorical analyses.

### Longitudinal analysis

The level of adherence to the EAT-Lancet diet was associated with cognitive decline, with a 0.05 unit (95% CI 0.02, 0.08; *P* < 0.01) slower rate in global cognitive decline per SD (3.7 point) increase in EAT-Lancet diet (Table 3). This equals being 4.5 years younger in age per SD increase in EAT-Lancet diet score. Similarly, comparing tertiles of adherence, individuals with highest adherence to the EAT-Lancet diet showed slower cognitive decline compared to individuals with lowest adherence (difference T1 versus T3 [95% CI]: 0.12 [0.05, 0.20], *P* < 0.01).

**Table 3:** Regression output association EAT-Lancet index and cognitive change (longitudinal)

	Crude model	Model 1	Model 2
Global cognition			
Tertile 1	REF	REF	REF
Tertile 2	0.06 [−0.01, 0.14] 0.08	0.07 [−0.01, 0.14] 0.07	0.06 [−0.02, 0.13] 0.14
Tertile 3	0.13 [0.05, 0.20] <0.01	0.13 [0.06, 0.21] <0.01	0.12 [0.05, 0.20] <0.01
Continuous	0.05 [0.02, 0.08] <0.01	0.05 [0.02, 0.08] <0.01	0.05 [0.02, 0.08] <0.01
Episodic memory			
Tertile 1	REF	REF	REF
Tertile 2	0.01 [−0.15, 0.16] 0.94	0.01 [−0.15, 0.18] 0.87	−0.01 [−0.17, 0.16] 0.93
Tertile 3	0.10 [−0.06, 0.25] 0.21	0.10 [−0.06, 0.26] 0.21	0.08 [−0.08, 0.24] 0.32
Continuous	0.03 [−0.03, 0.09] 0.35	0.03 [−0.04, 0.09] 0.42	0.02 [−0.05, 0.08] 0.58
Attention and working memory			
Tertile 1	REF	REF	REF
Tertile 2	0.05 [−0.12, 0.21] 0.57	0.01 [−0.17, 0.18] 0.94	−0.01 [−0.18, 0.17] 0.94
Tertile 3	0.32 [0.15, 0.48] <0.01	0.28 [0.11, 0.46] <0.01	0.27 [0.10, 0.45] <0.01
Continuous	0.12 [0.05, 0.19] <0.01	0.11 [0.04, 0.18] <0.01	0.11 [0.04, 0.18] <0.01
Information processing speed			
Tertile 1	REF	REF	REF
Tertile 2	0.10 [−0.03, 0.24] 0.13	0.11 [−0.02, 0.25] 0.11	0.10 [−0.04, 0.24] 0.15
Tertile 3	0.08 [−0.05, 0.22] 0.21	0.08 [−0.05, 0.22] 0.24	0.06 [−0.07, 0.20] 0.35
Continuous	0.03 [−0.02, 0.09] 0.21	0.03 [−0.03, 0.08] 0.30	0.02 [−0.03, 0.08] 0.46
Executive functioning			
Tertile 1	REF	REF	REF
Tertile 2	0.17 [0.05, 0.30] 0.01	0.17 [0.04, 0.30] 0.01	0.16 [0.02, 0.29] 0.02
Tertile 3	0.17 [0.05, 0.29] 0.01	0.15 [0.02, 0.27] 0.03	0.13 [0.00, 0.26] 0.06
Continuous	0.09 [0.04, 0.13] <0.01	0.08 [0.02, 0.13] <0.01	0.07 [0.02, 0.12] 0.01

Crude model: adjusted for baseline cognition score; Model 1: additionally adjusted for age, gender, education and ApoE4 carrier status; Model 2: additionally adjusted for BMI, physical activity, smoking and alcohol consumption. Data are β [95% CI] *P*-value. In the continuous analysis, β is shown per SD = 3.7 points increment in EAT-Lancet index.

A similar pattern was observed with respect to the cognitive domains attention and working memory and executive functioning. Higher adherence to the EAT-Lancet diet was associated with slower rates of cognitive decline in attention and working memory (β per SD [95% CI]: 0.11 [0.04, 0.18], *P* < 0.01) and executive functioning (β per SD [95% CI]: 0.07 [0.02, 0.12], *P* = 0.01), equalling to being 9.7 and 4.4 years younger in age, respectively. For both cognitive domains, findings were confirmed in the categorical analysis. However, we did not find associations between the level of adherence to the EAT-Lancet diet with change in episodic memory and information processing speed.

## Discussion

In this cohort of cognitively healthy older adults, higher adherence to the EAT-Lancet diet was found to have a beneficial impact on cognitive ageing, manifested by associations with better global cognitive function and a slower rate of global cognitive decline. With respect to domain-specific cognitive functioning, individuals with a higher adherence to the EAT-Lancet diet showed better executive functioning and slower decline in this domain, as well as slower decline in attention and working memory. No associations were observed for episodic memory and information processing speed.

To the best of our knowledge, the association between the EAT-Lancet diet and cognitive ageing has previously been described in only one article [30]. In line with our findings, this article demonstrated that better adherence to the EAT-Lancet diet in midlife was associated with lower odds of poor cognitive function among Asian adults in later life. These findings are interesting, considering that the EAT-Lancet diet is more plant-based and thus more sustainable compared to other dietary patterns with demonstrated benefits for the ageing brain [31]. Further support that a shift towards more plant-based diets might positively impact cognition comes from research on the healthy plant-based diet index [32–34]. Interestingly, the addition of fish to a plant-rich diet was superior over a plant-rich dietary pattern without fish [32, 35], in line with the composition of the EAT-Lancet diet.

Previous research on the EAT-Lancet diet in relation to other health outcomes with shared aetiology as cognitive ageing further substantiates our findings. Higher adherence to the EAT-Lancet diet has been associated with lower risk of type 2 diabetes in three European cohorts [5–7], though not in a cohort of Mexican women [36]. In addition, the EAT-Lancet diet seems beneficial for cardiovascular health as higher adherence to the diet has been associated with lower risk of coronary events [8], cardiovascular disease [10], subarachnoid stroke [37], and improved cardiometabolic markers including blood pressure and cholesterol [9]. However, two studies showed a null association with cardiovascular outcomes [12, 38]. Finally, higher scores on the EAT-Lancet diet were associated with favourable obesity outcomes, as measured by BMI [12] and waist circumference [11].

From the perspective of nutrient composition, a positive link between the EAT-Lancet diet and cognitive ageing is conceivable. The EAT-Lancet diet is plant-focused and delivers various plant nutrients including carotenoids, polyphenols, vitamin E, and some B-vitamins. These nutrients have anti-inflammatory and anti-oxidant properties, via which they can support healthy brain ageing [39]. Importantly, the EAT-Lancet diet also recommends consumption of fish and does not completely restrict consumption of other animal foods, thus providing the brain with omega-3 fatty acids and vitamin B_12_. Omega-3 fatty acids contribute to healthy brain ageing due to their anti-inflammatory and vascular health promoting properties. In addition, they are an important structural component of the brain [40]. Vitamin B_12_, together with folic acid and vitamin B_6_, prevent homocysteine accumulation and thereby help lower the risk of dementia [41].

However, while the EAT-Lancet diet does contain all nutrients required for healthy brain ageing, concerns have been raised if the diet supplies some of these nutrients in adequate amounts.

The first nutrient of concern is vitamin B_12_. According to the original EAT-Lancet diet publication, adopting the EAT-Lancet diet would improve intake of all nutrients but vitamin B_12_ [3]. Similarly, a nutrient adequacy analysis confirmed that the EAT-Lancet diet does not provide enough vitamin B_12_, with estimated intake being 93% of recommended nutrient intake [42]. Vitamin B_12_ is not only of great importance for brain health, but also for bone health [43]. The lack of vitamin B_12_ is especially of concern for the ageing population, as vitamin B_12_ absorption decreases with age and can be compromised with use of certain medications [44].

Secondly, the calcium content of the EAT-Lancet diet may be inadequate, with the estimated intake being 86% of the recommended nutrient intake [42]. Even though calcium is not considered a nutrient of prime interest for the ageing brain, this nutrient is essential for the bones and in the prevention of osteoporosis and fractures [45]. Adequate intake of calcium is especially relevant in the context of a plant-based diet high in phytates, as this anti-nutrient may decrease the absorption of calcium. Fortunately, this is particularly the case in non-balanced diets low in minerals but high in phytate, and may be less relevant when adhering to a balanced diet such as the EAT-Lancet diet [46].

Finally, the protein and amino acid content of the diet may be a point of concern, especially in the light of the increase in protein requirements with ageing [47]. The EAT-Lancet diet recommends largely replacing animal by plant protein, despite the lower protein quality (e.g. suboptimal essential amino acid content) of plant foods compared to animal foods [47]. While it is possible to consume a plant-based diet high in protein with a complete amino acid profile, it requires knowledge on protein content of plant foods and how to combine these protein sources. The possible deficit of protein is particularly of relevance for the muscle, as adequate consumption of high-quality protein is crucial to prevent age-related decline in muscle mass and strength [47].

Further research is needed to investigate if the EAT-Lancet diet sufficiently supplies the ageing population with these nutrients of concern, and to find out how adherence to the EAT-Lancet diet relates to other age-related outcomes. For now, caution is warranted before advising older adults to adopt the EAT-Lancet diet. Intake and/or status of nutrients at risk should be monitored, and possibly supplementation or consumption of fortified foods may be required. Furthermore, it is crucial that older adults are educated on how to create balanced high protein plant-based meals that cover amino acid needs.

Finally, each study has limitations that should be considered. An important limitation of the current study is that the variation in dietary intake between participants was small. This is evidenced by the narrow range of EAT-Lancet diet scores in the middle tertile of adherence and happened despite the extensive dietary assessment. The low variation in scores on the EAT-Lancet diet may explain why some result were only significant in the continuous, but not in the categorical analyses. Regardless, continuous results should be prioritised over categorical results. Another limitation includes the 2-year duration of follow-up, which is a relatively short period to capture cognitive deteriorations in older adults without cognitive complaints. Nonetheless, the use of an extensive cognitive test battery as a sensitive method to pick up cognitive differences allowed us to demonstrate associations.

In conclusion, we demonstrated a beneficial association between better adherence to the EAT-Lancet diet with cognitive ageing in cognitively healthy older adults. It is too early, however, to recommend the ageing population to adopt the EAT-Lancet diet, as the diet may provide inadequate amounts of several nutrients of major importance to healthy ageing. Further research focussing on other age-related conditions would be required to verify its relevance to the ageing population.

## Supplementary Material

aa-23-1942-File002
