# Supplementary material for: The association between adherence to the EAT-Lancet diet and cognitive ageing
Source: Age Ageing. 2024 May 15;53(Suppl 2):ii39–46. doi: 10.1093/ageing/afae032 (PMC11094393; doi:10.1093/ageing/afae032)
Supplement: aa-23-1942-File002 [file aa-23-1942-file002.docx]

**The association between adherence to the EAT-Lancet diet and cognitive ageing.**

**Supplemental materials:**

**Supplementary table 1** Overview of food items constituting the 14 EAT-Lancet diet food groups.

**Supplementary table 2** Description of cognitive tests.

**Supplementary table 3** Nutrient intake of the B-proof study population according to EAT-Lancet index tertile.

**Supplementary table 4** Overview of food components and portion sizes for the EAT-Lancet, MIND and Mediterranean diet.

**Supplementary table 1**: Overview of food items constituting the 14 EAT-Lancet diet food groups.

| Emphasized food components | | |
| --- | --- | --- |
|  | Vegetables | Cauliflower, broccoli and other cabbages, spinach, beets, endive, green beans, other cooked vegetables, lettuce, raw endive, other raw vegetables |
|  | Fruits | Apple, banana, orange, strawberry, other fruits |
|  | Unsaturated oils | Olive oil, dressing based on oil, other oils |
|  | Legumes | Legumes, soy products |
|  | Nuts | Peanut butter, peanuts, cocktail nuts, walnuts, mixed nuts, other nuts and seeds |
|  | Whole grains | Whole grain breakfast cereal, cooked oatmeal, wheat porridge, whole grain rusk, whole grain crispbread, rye bread, whole grain bread, whole grain pasta, brown rice, bulgur, millet, couscous |
|  | Fish | Shellfish, mussels, flounder, trout, herring, salmon, other types of fish |
| Limited intake food components | | |
|  | Beef and lamb* | Beef, lamb, beef/lamb liver, beef minced meat, organ meats, beef fried meat snacks |
|  | Pork* | Pork, pork liver, bacon, pork minced meat, smoked sausage, pork fried meat snacks |
|  | Poultry* | Chicken, turkey, chicken fried meat snacks |
|  | Eggs | Eggs |
|  | Dairy | Milk, buttermilk, chocolate milk, yoghurt, custard, drink breakfast, cheese, cream, ice cream |
|  | Potatoes | Fries, cooked and baked potatoes, mashed potatoes |
|  | Added sugar | Calculated based on added sugar composition database by Sluik and colleagues |

* Some meat products could not be classified into one component. To this end, we included meat products from other animal origin (horse, hare) for 50% in the beef and lamb component, and for 50% in the pork component. The same approach was taken for meat products with unknown origin and meat products containing both beef and pork in equal amounts (i.e. half-half minced meat). Finally, meat products containing both beef and pork but consisting primarily (≥75%) out of either beef or pork, have been assigned to the respective group.

**References**

Sluik D, van Lee L, Engelen AI, Feskens EJM. Total, free, and added sugar consumption and adherence to guidelines: The dutch national food consumption survey 2007–2010. Nutrients. [Article]. 2016;8(2).

**Supplementary table 2**: Description of cognitive tests.

| Domain | Test | Description | Scoring |
| --- | --- | --- | --- |
| Episodic memory | RAVLT immediate | Recall of 15 words in five trials | 0-45 |
|  | RAVLT delayed | Delayed recall of the 15 words after 20 minutes | 0-15 |
|  | RAVLT recognition | Recognition of the 15 words in a list of 30 words | 0-30 |
| Attention and working memory | Digit span forward | Recall of digit sequences with increasing length in forward order | 0-9 |
|  | Digit span backward | Recall of digit sequences with increasing length in backward order | 0-8 |
| Information processing speed | Stroop part I and II | Naming colour words written in black ink (part I) and coloured blocks (part II) as fast as possible.  Outcome is mean part I and II | 0 – ∞ s |
|  | Trail making test part A | Draw lines connecting numbers in chronological order as fast as possible | 0 - 300 s |
|  | SDMT | Match symbols with digits within 90 s as fast as possible. | 0 - 110 |
| Executive functioning | Stroop interference | Naming colour words written in black ink (part I), coloured blocks (part II) and colour words written in an incongruent colour ink (part III) Outcome is part III corrected by parts I and II | 0 – ∞ |
|  | Trail making test part B/A | Draw lines connecting numbers in chronological order (part A) or numbers and letters alternating in chronological and alphabetical order (part B). Outcome is ratio part B/A | 0-300 s |
|  | Letter fluency | Name as many words as possible starting with a specific letter in 60 s | 0 - ∞ |

Abbreviations: Rey Auditory Verbal Learning Test (RAVLT); Symbol Digit Modalities Test (SDMT)

Table is reused from supplementary information from van Soest et al. (2023).

**References**

van Soest APM, van de Rest O, Witkamp RF, et al.; The association between adherence to a plant-based diet and cognitive ageing. *European Journal of Nutrition* 2023;**62**(5):2053-2062. doi: 10.1007/s00394-023-03130-y.

**Supplementary table 3**: Nutrient intake of the B-proof study population according to EAT-Lancet index tertile.

| **Nutrient** | **Overall  (n=630)** | **Low  (n=241)** | **Middle  (n=203)** | **High  (n=186)** | **p-value** |
| --- | --- | --- | --- | --- | --- |
|  |  |  |  |  |  |
| Energy (kCal) | 1948 ± 508 | 1932 ± 506 | 2010 ± 527 | 1899 ± 487 | 0.09 |
| Protein (g) | 73 ± 18 | 73 ± 16 | 76 ± 18 | 71 ± 18 | <0.01 |
| Protein, animal origin (g) | 45 ± 13 | 48 ± 12 | 47 ± 13 | 40 ± 13 | < 0.001 |
| Protein, plant origin (g) | 28 ± 8 | 25 ± 7 | 29 ± 9 | 31 ± 8 | < 0.001 |
| Carbohydrates (g) | 214 ± 63 | 210 ± 62 | 224 ± 68 | 208 ± 56 | 0.02 |
| Sugar (g) | 111 ± 40 | 110 ± 42 | 117 ± 43 | 106 ± 35 | 0.02 |
| Starch (g) | 103 ± 32 | 100 ± 32 | 107 ± 34 | 101 ± 29 | 0.08 |
| Fibre (g) | 23 ± 7 | 21 ± 6 | 25 ± 7 | 25 ± 7 | < 0.001 |
| Fat (g) | 78 ± 27 | 79 ± 28 | 79 ± 27 | 76 ± 27 | 0.42 |
| Cholesterol (mg) | 202 ± 79 | 230 ± 89 | 199 ± 71 | 168 ± 58 | < 0.001 |
| SFA (g) | 28 ± 11 | 31 ± 13 | 28 ± 10 | 25 ± 8 | < 0.001 |
| MUFA (g) | 26 ± 10 | 26 ± 10 | 27 ± 10 | 26 ± 11 | 0.87 |
| PUFA (g) | 16 ± 8 | 14 ± 7 | 16 ± 8 | 17 ± 9 | < 0.001 |
| Alcohol (g) | 14 ± 14 | 13 ± 14 | 14 ± 12 | 15 ± 15 | 0.34 |
| linoleic acid (g) | 13 ± 7 | 12 ± 6 | 14 ± 7 | 14 ± 8 | < 0.001 |
| α-linolenic acid (g) | 1.3 ± 0.8 | 1.2 ± 0.6 | 1.3 ± 0.8 | 1.4 ± 0.9 | < 0.001 |
| EPA (g) | 0.07 ± 0.08 | 0.05 ± 0.05 | 0.08 ± 0.09 | 0.10 ± 0.10 | < 0.001 |
| DHA (g) | 0.11 ± 0.11 | 0.08 ± 0.07 | 0.12 ± 0.12 | 0.14 ± 0.14 | < 0.001 |
| Vitamin B12 (mg) | 4.1 ± 2.0 | 4.0 ± 1.8 | 4.4 ± 2.3 | 3.8 ± 1.9 | 0.01 |
| Folic acid (mcg) | 188 ± 56 | 173 ± 51 | 200 ± 59 | 193 ± 54 | <0.001 |

Data are mean ± SD. Abbreviations: SFA: saturated fatty acids, MUFA: monounsaturated fatty acids, PUFA: polyunsaturated fatty acids, EPA: eicosapentaenoic acid, DHA: docosahexaenoic acid.

| **EAT-Lancet diet^1^** | | | **MIND diet^2^** | | **Mediterranean diet^3^** | |
| --- | --- | --- | --- | --- | --- | --- |
| **Components** | **Portion size (grams)** | **Portion size (portion)^4^** | **Components** | **Portion size (portion)** | **Components** | **Portion size (portion)** |
| Vegetables | >300 g/d | >6/d | Green leafy vegetables | ≥ 6/w | Vegetables | >4/d |
|  |  |  | Other vegetables | ≥ 1/d |  |  |
| Fruits | >200 g/d | >2/d | Berries | ≥2/w | Fruits | >3/d |
| Unsaturated oils | >40 g/d | >4/d | Olive oil | Primary oil | Olive oil | ≥1/d |
|  |  |  | Butter, margarine | < T/d |  |  |
| Legumes | >75 g/d | >1.5/d | Beans | >3/w | Legumes, nuts & beans | >6/w |
| Nuts | >50 g/d | >2/d | Nuts | ≥5/w |  |  |
| Whole grains | >232 g/d | >5/d | Whole grains | ≥ 3/d | Non-refined grains | >4/d |
| Fish | >28 g/d | >2/w | Fish | >1/w | Fish | >6/w |
| Beef and lamb | <7 g/d | <0.5/w | Red meat and products | <4/w | Red meat and products | ≤1/w |
| Pork | <7 g/d | <0.5/w |  |  |  |  |
| Poultry | <29 g/d | <2/w | Poultry | ≥2/w | Poultry | ≤3/w |
| Eggs | <13 g/d | <2/w |  |  |  |  |
| Dairy | <250 g/d | <2/d | Cheese | <1/w | Full-fat diary | ≤10/w |
| Potatoes | <50 g/d | <0.5/d |  |  | Potatoes | >2/d |
| Added sugar | <31 g/d | n/a | Pastries, sweets | < 5/w |  |  |
|  |  |  | Fast/fried foods | <1/w |  |  |
|  |  |  | Wine | 1/d | Alcohol | <300mL/d but >0 |

**Supplementary table 4**: Overview food components and portion sizes for the EAT-Lancet, MIND and Mediterranean diet.

Green colour indicates emphasized food components, orange colour indicates limited intake food components.

**References**

^1^ Based on Stubbendorff, A., Sonestedt, E., Ramne, S., Drake, I., Hallström, E., & Ericson, U. (2022). Development of an EAT-Lancet index and its relation to mortality in a Swedish population. *The American journal of clinical nutrition*, *115*(3), 705-716.

^2^ Based on Morris, M. C., Tangney, C. C., Wang, Y., Sacks, F. M., Bennett, D. A., & Aggarwal, N. T. (2015). MIND diet associated with reduced incidence of Alzheimer's disease. *Alzheimer's & Dementia*, *11*(9), 1007-1014.

^3^ Based on Panagiotakos, D. B., Pitsavos, C., Arvaniti, F., & Stefanadis, C. (2007). Adherence to the Mediterranean food pattern predicts the prevalence of hypertension, hypercholesterolemia, diabetes and obesity, among healthy adults; the accuracy of the MedDietScore. *Preventive medicine*, *44*(4), 335-340.

^4^ Converted based on https://portie-online.rivm.nl/
